# A density functional theory study of the role of functionalized graphene particles as effective additives in power cable insulation

**DOI:** 10.1098/rsos.170772

**Published:** 2018-02-07

**Authors:** Shuwei Song, Hong Zhao, Xiaonan Zheng, Hui Zhang, Yang Liu, Ying Wang, Baozhong Han

**Affiliations:** 1 Key Laboratory of Engineering Dielectrics and Its Application, Harbin University of Science and Technology, Harbin, 150080, People's Republic of China; 2 MIIT Key Laboratory of Critical Materials Technology for New Energy Conversion and Storage, School of Chemistry and Chemical Engineering, Harbin Institute of Technology, Harbin, 150080, People's Republic of China; 3 State Key Laboratory of Rare Earth Resource Utilization, Changchun Institute of Applied Chemistry, Chinese Academy of Sciences, Changchun, 130022, People's Republic of China; 4 Shanghai Qifan Cable Co. Ltd, Shanghai, 200008, People's Republic of China

**Keywords:** graphene-based additives, power cable insulation, density functional theory, interfacial interaction

## Abstract

The role of a series of functionalized graphene additives in power cable insulation in suppressing the growth of electrical treeing and preventing the degradation of the polymer matrix has been investigated by density functional theory calculations. Bader charge analysis indicates that pristine, doped or defect graphene could effectively capture hot electrons to block their attack on cross-linked polyethylene (XLPE) because of the π–π conjugated unsaturated structures. Further exploration of the electronic properties in the interfacial region between the additives and XLPE shows that N-doped single-vacancy graphene, graphene oxide and B-, N-, Si- or P-doped graphene oxide have relatively strong physical interaction with XLPE to restrict its mobility and rather weak chemical activity to prevent the cleavage of the C–H or C–C bond, suggesting that they are all potential candidates as effective additives. The understanding of the features of functionalized graphene additives in trapping electrons and interfacial interaction will assist in the screening of promising additives as voltage stabilizers in power cables.

## 1. Introduction

Cross-linked polyethylene (XLPE) insulation materials have been extensively used for high-voltage cables in electrical networks [1–3]. However, electrical treeing and space charge accumulation are main obstacles for the lifetime and voltage rating of power cables [4–9]. Continued efforts were made on understanding the initiation mechanisms [4] and increasing resistance to electrical treeing [10–16]. Yi *et al.* modified XLPE with a small amount of chlorinated polyethylene and revealed the role of the polar group in increasing the trap density to attract the free charge carriers [16]. Besides the modification of the XLPE itself, the addition of a voltage stabilizer is another promising method for improving the electrical treeing resistance [10–12,17–22]. The voltage stabilizer works as an additive to capture the high-energy electrons and prevent the degradation of the polymer matrix. Jarvid *et al.* investigated the stabilization effect of benzyl-type organic compounds through ramp experiments and found that they are highly effective in raising the inception level of electrical treeing and further inhibiting the formation of electrical treeing [12].

Recently, progress has been made towards the wide experimental observation of nanosize particle fillers in suppressing the growth of electrical treeing, such as SiO_2_, MgO and ZnO [23–31], together with the analysis and explanation for the potential role of nanocomposite additives [32–34]. As is well known, the addition of nanoparticles has a major influence on the electronic properties of the interfacial region between them and the polymer matrix, which could result in the charge redistribution of the system or probably be accompanied by cleavage and formation of a chemical bond. So the study of the interfacial interaction will be very necessary to understand the essential role of additives in power cable insulation. In our previous study, we performed quantum chemical molecular dynamics simulations to reveal the role of the SiO_2_ nanocluster as a stabilizer in trapping electrons, preventing the cleavage of C–C bond and restraining the mobility of polyethylene chains by hydrogen bonds [31]. Inspired by this, it will be significantly informative to predict theoretically the advantages and disadvantages of some potential nanoparticle fillers in hindering electrical treeing formation, and further guide the rational design of and screening for prospective nanoparticle fillers.

Graphene, as one of the most popular two-dimensional materials, has been widely applied in the fields of photocatalysis, pharmacology, solar cells etc. [35–41]. The functionalization of graphene by introducing various heteroatoms or defects has also been confirmed to be an effective method for improving the activity of graphene [41–44]. The feasibility of graphene or functionalized graphene nanoparticles as potential additives in power cable insulation has been reported experimentally in recent years. By making use of the pulse electroacoustic technique, Mancinelli and co-workers found that the addition of graphene oxide as a filler in low-density polyethylene could remarkably reduce space charge accumulation and consequently lead to a smaller electric field distortion [45,46]. Lei *et al.* prepared graphene-enhanced low-density polyethylene by pretreating and the melt-compounding method, and revealed its thermal stability and improved mechanical properties by scanning electron microscopy, Raman spectra etc. [47]. Bu *et al.* explored the advancement of antioxidant functionalized graphene oxide filler in promoting the thermal stability and retaining the electrical insulating properties of low-density polyethylene [48]. However, no theoretical studies are reported for the particular role of graphene-family fillers in power cable insulation. Therefore, in the present study, we performed first-principle calculations to investigate the electronic properties in the interfacial region between a series of graphene-based materials and the polymer matrix with the purpose of evaluating the performance of graphene-based fillers in power cable insulation.

## 2. Simulation methods and models

### 2.1. Simulation methods

Spin-polarized density functional theory (DFT) calculations with the Perdew–Burke–Ernzerhof (PBE) functional [49] were performed by using Vienna Ab initio Simulation Package (VASP) [50,51]. The projected–augmented wave (PAW) method was used to compute electronic structures of the systems [52,53]. The energy and force convergence criteria for geometry optimization were set to be 1.0 × 10^−5^ eV and 0.05 eV Å^−1^, respectively. The wave function at each *k* point was expanded in plane wave basis sets with a kinetic cut-off energy of 450 eV. The integration of the Brillouin zone was conducted with a 2 × 2 × 1 Monkhorst–Pack grid centred at *Γ*-point. For minimizing periodic interactions, a vacuum layer as large as 15 Å was set in the perpendicular direction. The DFT-D2 approach [54] was performed to estimate the van der Waals interaction in the geometry optimizations and energy calculations. Bader charge analysis [55] was employed to obtain the quantitative description of the charge distribution. The climbing-image nudged elastic band (CI-NEB) [56] method was used to locate the transition state and determine the minimum energy pathway by constructing five intermediate images between the initial (reactant) and final (product) structures. For characterizing the nature of the transition state more accurately, the CI-NEB calculations by inserting seven intermediate images combined with the harmonic vibrational frequency analysis were performed for the representative candidates SVG and N-SVG, and the results showed that they are quite consistent with these by inserting five images.

### 2.2. Simulation models

**Figure 1.**
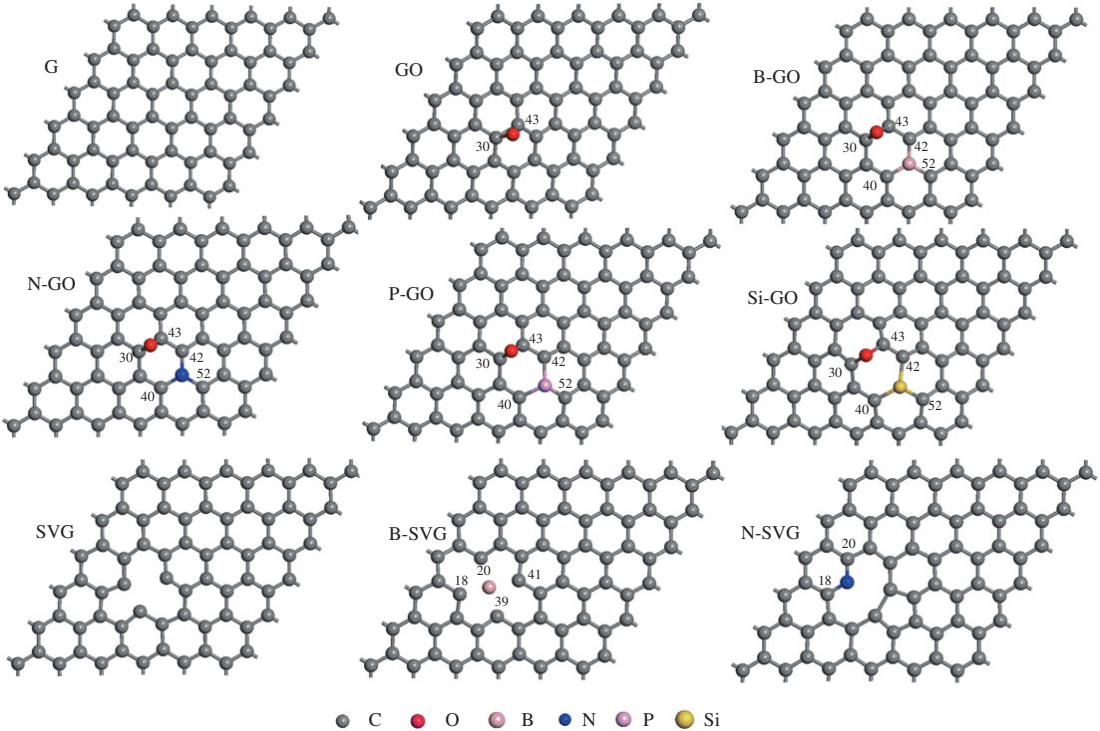
The supercell models of pristine and functionalized graphene sheets.

A (6 × 6) supercell was constructed to model the pristine graphene sheet (G). Based on it, models of graphene oxide (GO), GO doped with B, N, Si or P atoms (B-GO, N-GO, Si-GO, P-GO), as well as single-vacancy graphene (SVG) and SVG doped with B or N atoms (B-SVG, N-SVG) were constructed and optimized, as shown in figure 1. The oxide or heteroatom-doping configurations and the content of doped atoms in the functionalized graphene mentioned above are popular and extensively reported by experimental and theoretical studies [42,57–62]. The chain-like 4-methylheptane C_8_H_18_ (i.e. CH_3_CH_2_CH_2_CH(CH_3_)CH_2_CH_2_CH_3_) organic molecule was used to simulate the chemical activity of the polyethylene chain on the surface of the graphene series sheet. The nearest distance of two C_8_H_18_ molecules in the neighbouring lattice is more than 5.6 Å so that the weak van der Waals interaction between them can be reasonably ignored. The present theoretical model is mainly concerned with the simulation of C–H bond-breaking behaviour of C_8_H_18_ by the catalytic action of graphene series, and the local interaction between them, which could effectively reflect the main interaction between the XLPE and graphene fillers in the real physical problem.

## 3. Results and discussion

As predicted, the addition of nanosize particle fillers in power cable insulation was able to change the charge distribution and produce the interfacial interaction with XLPE. Therefore, the role of graphene-based series additives will be discussed below with respect to their abilities of trapping hot electrons, restricting the mobility of the polyethylene chains and preventing the C–H or C–C bond cleavage of XLPE.

### 3.1. Trapping hot electrons

In general, the charge carriers are produced from the system or injected from the electrode. If the nanosized particles existing in the system could constrain the flow of charge carriers by accumulating electrons on their surfaces, the resulting negative charge will make the electric field of the cathode weaker and then suppress the continued injection of electrons from the cathode, so as to reduce the amount of space charge in the system.

**Figure 2.**
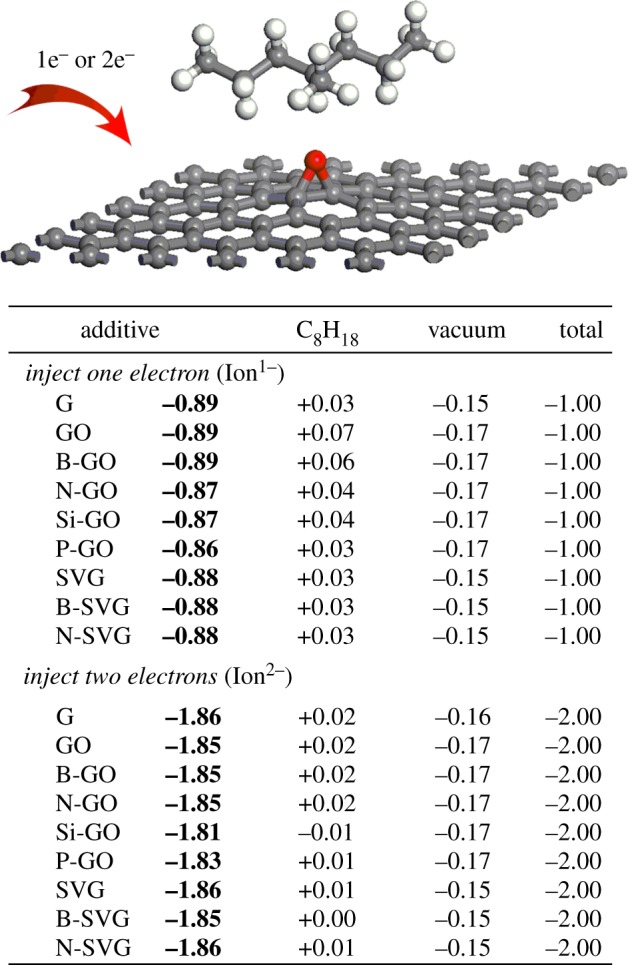
Bader charge analysis when 1 or 2 extra electrons are, respectively, injected to the systems.

Figure 2 shows the Bader charge analysis of a C_8_H_18_ molecule adsorbed upon a series of functionalized graphene surfaces. When 1 or 2 extra electrons are injected into the systems, denoted as the Ion^1−^ and Ion^2−^ case, respectively, the charge is redistributed among the atoms. By summarizing the charge of atoms belonging to the C_8_H_18_ molecule and graphene series, respectively, we could determine the location of extra electrons in the systems. One can see that the charge values at the graphene series are up to −0.89 and −1.86 for the Ion^1−^ and Ion^2−^ case, respectively, whereas those at the C_8_H_18_ molecule are close to zero. Thus the electron density is mainly concentrated on the graphene series, not on the C_8_H_18_ molecule. The strong ability of capturing electrons is mainly generated by the π–π conjugated unsaturated structures of the graphene series, which is consistent with a previous report for polycyclic additive [22]. It suggests that the graphene-based particle fillers have the ability of trapping electrons, thereby protecting the XLPE against the attack of hot electrons. Additionally, it is found that the summarized charge values for the pristine graphene and functionalized (defect or doped) graphene are almost the same for each Ion^1−^ and Ion^2−^ case, which indicates that they have almost equal ability to trap electrons. So we could infer that the graphene-based particles, which include not only the nine patterns in this study but also other defect or doped graphene, are all very good candidates as additives for power cable insulation in the general sense of effectively trapping hot electrons to suppress the growth of electrical treeing. The mechanism of using unsaturated electronic structures of additives to trap extra electrons could extend to more broad cases. It could help us to understand why most of the nanosize particles could act as good stabilizers, because they usually have unsaturated surface structures with similar properties to the graphene series we studied.

### 3.2. Interfacial interaction

#### 3.2.1. Physical interaction

The interaction between the additive and XLPE in the interfacial region is very important for evaluating the mobility and stability of the polyethylene chains. In the present theoretical models, the interaction of the additive with the XLPE could be evaluated by the adsorption ability of the graphene series to the C_8_H_18_ molecule. As typical two-dimensional materials, the pristine and functionalized graphene sheets could produce an adsorption effect for the C_8_H_18_ molecule, which could be seen from the calculated adsorption energies defined as follows:

$$E_{\mathrm{ads}}=E\text{(}{\text{C}}_{8}{\text{H}}_{18}\text{/graphene) }-E\text{(graphene)}-E\text{(}{\text{C}}_{8}{\text{H}}_{18}\text{),}$$

where *E*(C_8_H_18_/graphene) is the total energy of the pristine or functionalized graphene sheet with the adsorbed C_8_H_18_, and *E*(graphene) and *E*(C_8_H_18_) are the energies of pristine or functionalized graphene sheet and free C_8_H_18_ molecule, respectively.

**Figure 3.**
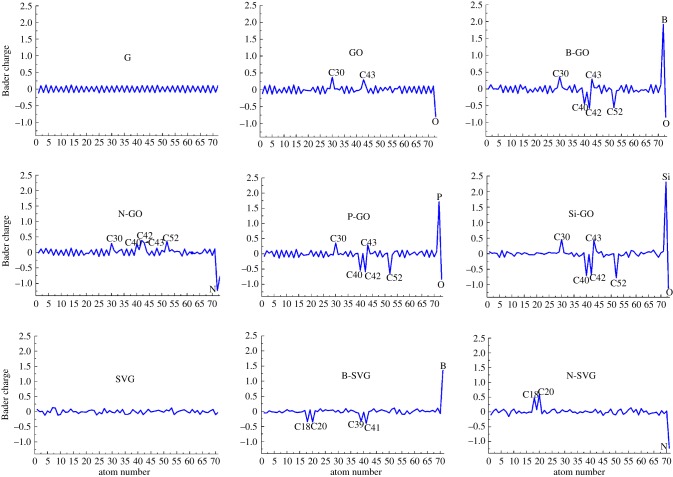
The Bader charge distribution at each atom of pristine and functionalized graphene sheets. The atoms with more charge are denoted, and their position in the supercell can be found in figure 1.

As listed in table 1, the calculated adsorption energies range from −0.16 to −0.49 eV. The adsorption distance is about 3.0 Å, suggesting the feature of weak physical adsorption through the van der Waals interaction. The adsorption ability increases in the order of G, GO and doped GO, SVG and doped SVG. The N-doped SVG has the strongest adsorption ability to C_8_H_18_ among the graphene-based models studied in the present work. As we know, heteroatom doping or defect in graphene will induce charge redistribution and increase dipole moment. The Bader charge distributions at each atom for all the nine patterns are depicted in figure 3. Because of the different electronegativities of C and B, N, Si or P dopants, the charge redistributes among the atoms and many active sites with big positive or negative charge values are produced in comparison with pristine graphene. The active sites with relatively large positive or negative charge all locate at the doped atom or the neighbouring carbon atoms, as shown in figure 3 and figure 1, which demonstrates the effect of doping. By taking GO as an example, the oxygen atom shows the highest negative charge density of −0.803 and the two carbon atoms connecting to the oxygen atom (C_30_, C_43_) possess highest positive charges of 0.374 and 0.302. The enhanced dipole–dipole interaction with C_8_H_18_ strengthens the adsorption ability. Stronger physical adsorption activity usually means a stronger ability to restrict the mobility of polyethylene chains. So the heteroatom doping and defect are effective functionalization ways to enhance the physical interaction of graphene-based additives and XLPE, and thus improve the stability of the XLPE.

**Table 1.** The adsorption energy (*E*_ads_), the energy barrier of H migration reaction (Δ*E*), as well as the energy difference between product and reactant (Δ*H*) for neutral and ionic states (in eV).

		Δ*E*			Δ*H*		
additive	*E*_ads_	neutral	Ion^1−^	Ion^2−^	neutral	Ion^1−^	Ion^2−^
G	−0.16	4.21	2.90	2.98	3.22	2.90	2.93
GO	−0.21	1.08	0.86	0.95	0.58	0.36	0.91
B-GO	−0.21	0.92	1.06	0.78	0.43	1.03	0.58
N-GO	−0.23	0.52	0.93	0.99	0.03	0.92	0.98
P-GO	−0.24	0.50	0.99	0.97	0.07	0.96	0.94
Si-GO	−0.26	1.64	0.79	0.91	1.18	0.37	0.88
SVG	−0.46	0.67	0.79	0.88	−0.23	−0.06	−0.01
B-SVG	−0.41	1.10	1.51	1.08	0.41	0.89	0.32
N-SVG	−0.49	1.63	2.18	1.89	1.15	1.95	1.37

#### 3.2.2. Chemical interaction

Besides the van der Waals interaction in the interfacial region discussed above, there may be stronger chemical interaction between the additive and XLPE. In the latter case, some chemical reactions can be involved, such as the H migration process. Owing to the doping or defect, there are some active sites in the functionalized graphene sheet as mentioned above, and these active sites probably possess the ability to attract the H atom of XLPE. As a following step, the H migration will probably induce the C–H or C–C bond cleavage of XLPE, and finally result in electrical treeing. Therefore, the reaction mechanism for H migration from C_8_H_18_ to the graphene-based sheets is studied with the conditions of neutral and anion (Ion^1−^ and Ion^2−^) states by injecting 0, 1 or 2 electrons to the systems, respectively. The transition states involved in the reaction pathways of H migration are located. Based on the energy barrier (Δ*E* = *E*_TS_ − *E*_R_) and the reaction energy (Δ*H* = *E*_P_ − *E*_R_), listed in table 1, we could judge how smoothly the H migration reaction takes places. If Δ*E* and Δ*H* are low, it means the H atom of XLPE could move easily to the graphene-based surface to build a new chemical bond with the atom in it, simultaneously resulting in the formation of a C_8_H_17_ radical. The radical is so active that it could cross-link with other carbon chain radicals, not beneficial for the stability of XLPE. On the other hand, the radical could react with other molecules in its environment, which will probably lead to the breaking of the C–C bond and the growth of electrical treeing. On the contrary, the graphene-based fillers with relatively high Δ*E* and Δ*H* for H migration reaction will be suitable additives to block H migration and further protect the XLPE.

**Figure 4.**
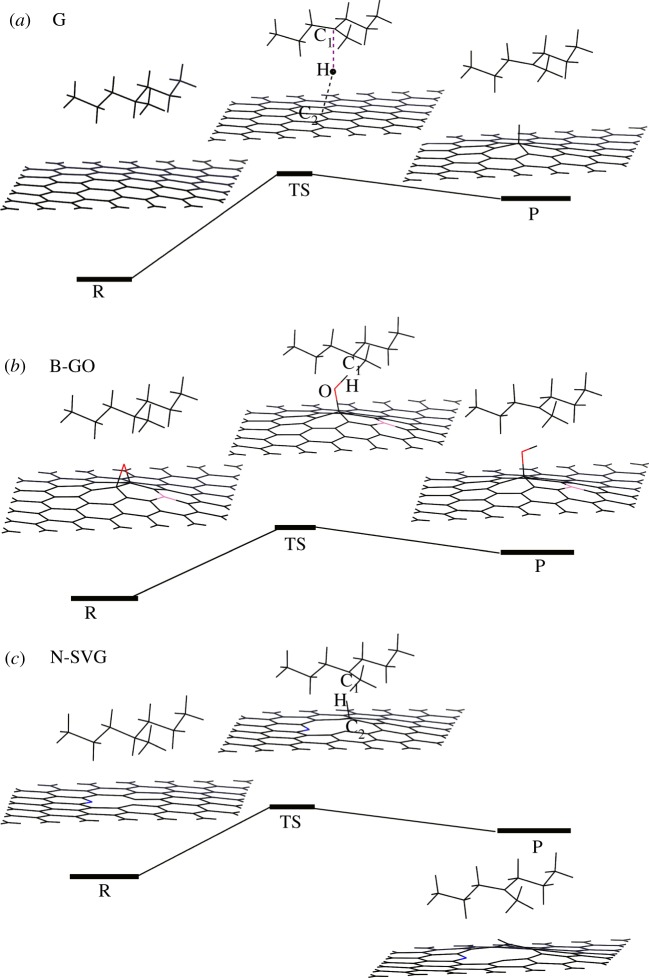
(*a*–*c*) The H migration reaction pathways including the reactant (R), transition state (TS) and product (P).

The geometrical structures of reactants, transition states and products on the H migration reaction pathways are shown in figure 4 by taking G, B-GO and N-SVG as representatives. It shows three kinds of typical H migration process. In figure 4*a*, the H atom in C_8_H_18_ transfers to the pristine graphene, forming a new C–H bond. In Figure 4*b*, the O–H bond is produced in the graphene oxide series (GO, B-GO, N-GO, Si-GO and P-GO). In figure 4*c*, the H atom prefers to bond with the C atom at the edge of the single-vacancy area of SVG, B-SVG and N-SVG. The key values of bond distances and angles for reactants, transition states and products on the H migration reaction pathways for neutral and ionic states can be seen in electronic supplementary material, table S1.

Owing to the highest barriers and reaction energies for both neutral and ionic states, as presented in table 1, H migration reaction to the pristine graphene (G) is mostly unfavourable. This is in agreement with our prediction that the new C–H bond will destroy the perfect π–π conjugated structure of the graphene sheet and make it less stable. In the cases of the graphene oxide series (GO, B-GO, N-GO, Si-GO, P-GO), the reaction barriers are around 1.0 eV, showing that the H migration reaction to the graphene oxide series is still difficult to proceed. Different from the cases above, the reaction barrier of SVG is reduced to be 0.67, 0.79 and 0.88 eV for the neutral, Ion^1−^ and Ion^2−^ state, respectively. In particular, the H migration products are more stable than the reactant as seen by the negative Δ*H* values for SVG in table 1. Both the lower barrier Δ*E* and negative Δ*H* indicate that SVG can promote H migration from the XLPE to the filler, and make the XLPE unstable. However, the circumstances are improved by B or N doping to SVG. Especially for N-SVG, the Δ*E* and Δ*H* increase to be more than 1.63 and 1.15 eV, respectively, which could effectively prevent the H migration process and protect the XLPE. Furthermore, it is worth noting that the Δ*E* and Δ*H* of ionic states are usually higher than those of the neutral state for most of the cases we have studied above. It tells us that the H migration reaction from XLPE to graphene-based fillers is even more difficult with the involvement of hot electrons, which is beneficial for protecting the XLPE. In summary, because of the relatively strong ability to hinder the H migration reaction and keep the stability of XLPE, the graphene-based particles studied in this work can be promising candidates as additives in power cable insulation, except SVG. Among all the considered potential candidates in this study, N-SVG could be the best one because of its strongest adsorption ability to XLPE (−0.49 eV) and relatively high barriers (more than 1.63 eV) and reaction energies (more than 1.15 eV) in the H migration reaction. In contrast with that, SVG without doped atoms is the weakest candidate as an additive in power cable insulation.

### 3.3. Insight into the design of promising additives in power cables

With the discussion and information above, we could have a better understanding of the role of graphene-based fillers in power cables. More importantly, it could guide the rational design of and screening for potential additives in power cable insulation. The strong ability to capture electrons is firstly essential as a property for a promising additive in power cables. By eliminating the attack of hot electrons, the growth of electrical treeing could be suppressed significantly. So the nanosized particles, graphene or nanotubes-based carbon materials, polycyclic organic molecules, as well as others with π–π conjugated structures are all potential candidates. The second factor which should be examined is the strong physical interaction between the additive and polyethylene to constrain the mobility of polyethylene chains. As calculated in this paper, the proper adsorption energies between them should be in the range −0.16 to −0.49 eV. It is also related to the electronic properties of the additives. Those polar particles with relatively big dipole moments usually show many advantages through the dipole–dipole interaction with XLPE. That is the reason that doped or defect graphene has stronger van der Waals interaction with XLPE than pristine graphene. Thirdly, the inert chemical activity of the additive in the interfacial region should be guaranteed, i.e. a high reaction barrier of around or more than 1.0 eV in the H migration reaction. If the particles have obvious dangling bonds or electron-defect atoms, such as SVG with dangling C or SiO_2_ with an exposed O atom, they will easily induce the H migration reaction and destroy the stability of XLPE. It is expected that the information above will be useful for the screening and development of promising additives in power cable insulation.

## 4. Conclusion

DFT calculations are performed to evaluate the role of pristine and functionalized graphene-based additives in power cable insulation. It is predicted that the graphene series or others with π–π conjugated unsaturated structures have a general ability of effectively trapping hot electrons to suppress the growth of electrical treeing. By calculating the adsorption energy and reaction pathway of H migration, it is found that N-doped single-vacancy graphene, graphene oxide, and B-, N-, Si- or P-doped graphene oxide possess relatively strong physical interaction with XLPE and quite weak chemical activity, which are promising additives to suppress electrical treeing and the degradation of the polymer matrix. The studies of the features of the trapping electrons, the physical interaction and chemical activity in the interfacial region provide further insights into the design of promising additives for power cables.

## Supplementary Material

Table S1

## Data accessibility

The datasets supporting this article have been uploaded as the electronic supplementary material.
