# Supplementary material for: A density functional theory study of the role of functionalized graphene particles as effective additives in power cable insulation
Source: R Soc Open Sci. 2018 Feb 7;5(2):170772. doi: 10.1098/rsos.170772 (PMC5830710; doi:10.1098/rsos.170772)
Supplement: Table S1 [file rsos170772supp1.doc]

**Electronic Supplementary Material**

**A density functional theory study of the role of functionalized graphene particles as effective additives in power cable insulation**

Shuwei Song, *^a^* Hong Zhao,*^a^** Xiaonan Zheng,*^b^* Hui Zhang,*^a^* Yang Liu,*^b^*^*^ Ying Wang, *^c^* and Baozhong Han*^a,d^**

*^a^* Key Laboratory of Engineering Dielectrics and Its Application, Harbin University of

Science and Technology, Harbin, 150080, PR China. Email: hongzhao@hrbust.edu.cn

*^b^* MIIT Key Laboratory of Critical Materials Technology for New Energy Conversion and Storage, School of Chemistry and Chemical Engineering, Harbin Institute of Technology, Harbin, 150080, PR China. Email: yang.liu@hit.edu.cn

*^c^* State Key Laboratory of Rare Earth Resource Utilization, Changchun Institute of

Applied Chemistry, Chinese Academy of Sciences, Changchun 130022, PR China.

*^d^*Shanghai Qifan cable Co., Ltd., Shanghai, 200008, PR China. Email: hbzhlj@163.com

**Table S1**: The key bond lengths (in Å) and angles (in degree) for reactants, transition states and products on the H immigration reaction pathways for neutral and ionic states.

| Additive | Parameter | Neutral | | | Ion^1−^ | | | Ion^2−^ | | |
| --- | --- | --- | --- | --- | --- | --- | --- | --- | --- | --- |
|  |  | R | TS | P | R | TS | P | R | TS | P |
| G | R(C_1_-H) | 1.108 | 2.974 | 3.845 | 1.104 | 2.875 | 2.875 | 1.104 | 2.275 | 2.827 |
|  | R(H-C_2_) | 4.031 | 2.868 | 1.128 | 2.636 | 1.130 | 1.130 | 2.635 | 1.144 | 1.141 |
|  | α(C_1_-H-C_2_) | 164.4 | 156.4 | 169.0 | 157.4 | 169.8 | 169.8 | 157.5 | 171.5 | 170.7 |
| GO | R(C_1_-H) | 1.102 | 1.436 | 2.597 | 1.103 | 1.477 | 2.723 | 1.107 | 1.713 | 2.336 |
|  | R(H-O) | 2.202 | 1.175 | 0.964 | 2.255 | 1.179 | 0.979 | 1.991 | 1.080 | 0.990 |
|  | α(C_1_-H-O) | 174.6 | 153.7 | 106.6 | 174.1 | 153.4 | 108.5 | 156.0 | 148.1 | 127.0 |
| B-GO | R(C_1_-H) | 1.103 | 1.443 | 2.619 | 1.101 | 1.617 | 2.255 | 1.108 | 1.583 | 2.522 |
|  | R(H-O) | 2.229 | 1.184 | 0.980 | 2.205 | 1.091 | 0.986 | 2.050 | 1.104 | 0.979 |
|  | α(C_1_-H-O) | 172.1 | 154.1 | 108.1 | 171.8 | 149.4 | 119.9 | 150.7 | 152.7 | 112.0 |
| N-GO | R(C_1_-H) | 1.101 | 1.359 | 2.430 | 1.103 | 1.705 | 2.233 | 1.107 | 1.718 | 2.066 |
|  | R(H-O) | 2.148 | 1.244 | 0.980 | 1.942 | 1.064 | 0.993 | 1.912 | 1.078 | 1.006 |
|  | α(C_1_-H-O) | 172.3 | 153.6 | 113.3 | 156.1 | 146.8 | 128.3 | 156.4 | 148.1 | 135.3 |
| Si-GO | R(C_1_-H) | 1.102 | 1.369 | 2.440 | 1.059 | 1.501 | 2.337 | 1.145 | 1.685 | 1.936 |
|  | R(H-O) | 2.275 | 1.247 | 0.982 | 2.302 | 1.199 | 0.985 | 1.936 | 1.101 | 1.023 |
|  | α(C_1_-H-O) | 169.0 | 156.3 | 123.7 | 166.1 | 155.2 | 130.6 | 149.7 | 151.8 | 145.3 |
| P-GO | R(C_1_-H) | 1.102 | 1.361 | 2.390 | 1.104 | 1.678 | 1.980 | 1.107 | 1.696 | 2.207 |
|  | R(H-O) | 2.252 | 1.254 | 0.984 | 2.048 | 1.108 | 1.015 | 1.950 | 1.077 | 0.993 |
|  | α(C_1_-H-O) | 167.6 | 155.7 | 125.6 | 153.9 | 151.7 | 141.9 | 154.2 | 150.6 | 132.9 |
| SVG | R(C_1_-H) | 1.106 | 1.406 | 2.734 | 1.105 | 1.448 | 2.720 | 1.105 | 1.482 | 2.719 |
|  | R(H-C_2_) | 2.422 | 1.369 | 1.075 | 2.429 | 1.354 | 1.078 | 2.392 | 1.357 | 1.078 |
|  | α(C_1_-H-C_2_) | 166.7 | 158.3 | 118.1 | 166.8 | 158.0 | 119.1 | 166.8 | 158.4 | 117.9 |
| B-SVG | R(C_1_-H) | 1.105 | 1.413 | 2.634 | 1.105 | 1.516 | 2.637 | 1.105 | 1.505 | 2.651 |
|  | R(H-C_2_) | 2.504 | 1.364 | 1.084 | 2.711 | 1.303 | 1.077 | 2.510 | 1.316 | 1.078 |
|  | α(C_1_-H-C_2_) | 173.4 | 160.1 | 124.4 | 166.7 | 158.4 | 114.8 | 173.5 | 158.3 | 115.4 |
| N-SVG | R(C_1_-H) | 1.103 | 1.742 | 2.973 | 1.104 | 2.148 | 2.962 | 1.104 | 1.831 | 3.030 |
|  | R(H-C_2_) | 2.442 | 1.231 | 1.080 | 2.588 | 1.149 | 1.081 | 2.585 | 1.218 | 1.081 |
|  | α(C_1_-H-C_2_) | 166.8 | 170.1 | 102.7 | 166.0 | 161.3 | 110.6 | 166.2 | 168.6 | 104.8 |

*The denotation of C_1_, C_2_, H, and O atoms are shown in Fig.4 of the main text.
